# The Use and Abuse of Condensed Milk and Patent Foods in Infant Feeding

**Published:** 1905-10-14

**Authors:** 


### THE USE AND ABUSE OF CONDENSED MILK AND PATENT FOODS IN INFANT FEEDING.

Dr. Still [1] does not condemn the use of condensed milk and patent foods, but recognises that most of these products have their virtues if only they be employed with an intelligent appreciation of their composition and the requirements of nature. It is, as he observes, impossible to retain in memory the actual composition of the innumerable articles of this kind which glut the market, but there are certain members of the group which are in sufficiently general use to command the attention of medical practitioners. These various foods fall into two classes, one class being intended for use as substitutes for fresh cow's milk, the other for use as an addition to cow's milk. The following classification certainly deserves to be committed to memory by all who engage in the treatment of children:—

*Class* 1.—(*a*) Condensed milk; (*b*) dried milk with cereal, the starch being completely converted—*e.g.*, Allenbury (Nos. 1 and 2), Horlick's malted milk; (*c*) dried milk with cereal, the starch being only partially converted—*e.g.*, Carnrick's soluble food, Milo food.

*Class* 2.—(*d*) Cereal, the starch being completely converted—Mellin's food, Hovis baby food No 1; (*e*) cereal, the starch being only partially converted—Allenbury No. 3, Benger's food, Savory and Moore's food; (*f*) cereal, the starch being mostly unconverted—Ridge's food, Neave's food, Frame food, Robb's biscuits, Chapman's wheat-flour.

It will be seen from this list that only those foods mentioned under the headings (*a*), (*b*), and (*d*) are free from starch. Dr. Still records his opinion, which will be generally shared, that all foods containing any unconverted starch whatever should be rigidly excluded from the dietary of an infant under the age of eight or nine months, and even then only introduced cautiously and with a sparing hand. He considers that even the small proportion of starch present in barley-water (say 1 to 2 per cent.) is often distinctly harmful. But this is not the only point at issue, for even though the starch be all converted, there exists in the patent foods above mentioned a proportion of carbohydrate largely in excess of that present in human milk. For instance, in the Allenbury foods (Nos. 1 and 2) the proportion of carbohydrate produced by dilution according to the directions is 10 to 11 per cent., or half as much again as the proportion present in human milk. Horlick's malted milk, again, when prepared according to official formula, yields a mixture containing nearly 12 per cent. of carbohydrates. This excess of carbohydrates may be well tolerated by some infants, but will in others certainly result in digestive and nutritional disturbances.

Dr. Still does well to insist upon the fact that the readiness with which a food is taken, and its apparent success at first, is no reliable criterion of its suitability as a diet for prolonged use. Foods containing an excess of carbohydrate often appear to achieve a great success early in their administration, yet later lead to rickets and its allies, probably, according to the author, by interfering with the due assimilation of fats. Dr. Still shares the general belief that the essential factor in the production of rickets is deficiency of fat, and these preparations, in addition to their assumed interference with fat assimilation, are themselves actually deficient in fats. Dr. Still asks the question, "What is to be considered a deficiency of fat?" and his answer is to the effect that 1.5 per cent. of fat is not sufficient to safeguard from rickets, that rickets may occur when the percentage is 2 or 2.5, and that no percentage less than 3 can be considered satisfactory for continued use. This fat deficiency in the milk substitutes upon the market is rendered evident by a glance at the published analyses of them. They show without exception a serious relative deficiency of fat and a large carbohydrate excess.

But Dr. Still, as has been said, finds a saving grace in these preparations when used with discretion. He refers to the commonly-heard experience that such and such a child "could not take fresh cow's milk," yet throve handsomely on condensed milk. The natural result of such an experience is that the parent pins her faith to the new discovery and perseveres in its use until the appearance of rickety manifestations drives her to the doctor. The probable explanation of the facts is well put by the author. The infant was probably tried in the first instance upon milk and water in the proportions of one to two, or even in equal parts. The result was vomiting or colic, and the resource was condensed milk, a teaspoonful in four, six, or even eight ounces of water. The following table illustrates the food value of such mixtures compared with that of the milk dilution previously attempted:—

| Condensed Milk | | Fresh Milk |
|---|---|---|
| 1 drachm to 4 ozs. | 1 drachm to 6 ozs. | 1 to 2 parts |
| Proteid, ·9 per cent. | ·6 per cent. | 1·2 per cent. |
| Fat, 1·1 „ „ | ·75 „ „ | 1·2 „ „ |
| Sugar, 4·5 „ „ | 3·2 „ „ | 1·3 „ „ (plus added sugar.) |

It is evident that the condensed milk in these mixtures is diluted to an extent which is rarely tried in the case of fresh cow's milk. The child has, in fact, responded to a diminshed dietary, and not to any special virtues in the digestibility of condensed milk. None the less, the use of condensed milk provides a ready means of achieving a high dilution without suggesting starvation diet to the mother; and if it were dispensed with when its purpose has been served, it would not sustain the evil reputation it must continue to bear in the minds of those engaged in the practice of children's hospitals.

Dr. Still is convinced that the proteid in the dried-milk preparations, and to a lesser degree in condensed milk, is easier of digestion than is that of fresh cow's milk. Peptonisation of cow's milk will reduce the disparity in this particular, but it is to be remembered that both peptonised and patent foods are competent to produce scurvy. The addition of sodium citrate to fresh cow's milk in the proportion of one grain to each ounce of milk, as suggested by Professor Wright, does not entail this objection. It reduces the indigestibility of the curd to a large extent, and should always be tried before resort is had to patent foods or peptonisation. Dr. Still concludes with a warning that all of us will do well to take to heart: "The doctor's orders, unless most carefully guarded and hedged about with cautions for each particular case, are apt to be taken as a general sanction for the particular food ordered. We may well, therefore, be careful in our advice, lest we should seem to countenance the indiscriminate use of these trade preparations, and thereby add to the deplorable suffering which such ignorant use brings upon infants. Of this I feel sure, that the more the medical man knows of the many simple methods of adapting fresh milk to the needs of the infant, the less use he will find for condensed milk and patent foods."

